# New Carrier Made from Glass Nanofibres for the Colorimetric Biosensor of Cholinesterase Inhibitors

**DOI:** 10.3390/bios8020051

**Published:** 2018-05-30

**Authors:** Lukáš Matějovský, Vladimír Pitschmann

**Affiliations:** 1 Faculty of Environmental Technology, University of Chemistry and Technology, Technická 5, 166 28 Prague, Czech Republic; 2 Oritest spol. s r.o., Nábřežní 90/4, 150 00 Prague, Czech Republic; pitschmann@oritest.cz

**Keywords:** biosensor, cholinesterase reaction, Ellman’s reagent, cellulose filter paper, glass fibre filter paper

## Abstract

Cholinesterase inhibitors are widely used as pesticides in agriculture, but also form a group of organophosphates known as nerve chemical warfare agents. This calls for close attention regarding their detection, including the use of various biosensors. One such biosensor made in the Czech Republic is the Detehit, which is based on a cholinesterase reaction that is assessed using a colour indicator—the Ellman’s reagent—which is anchored on cellulose filter paper together with the substrate. With the use of this biosensor, detection is simple, quick, and sensitive. However, its disadvantage is that a less pronounced yellow discoloration occurs, especially under difficult light conditions. As a possible solution, a new indicator/substrate carrier has been designed. It is made of glass nanofibres, so the physical characteristics of the carrier positively influence reaction conditions, and as a result improve the colour response of the biosensor. The authors present and discuss some of the results of the study of this carrier under various experimental conditions. These findings have been used for the development of a modified Detehit biosensor.

## 1. Introduction

Cholinesterase inhibitors are toxic substances that interfere with the cholinergic transfer mechanism of the nerve impulse. Carbamate and organophosphorus pesticides, or organophosphorus warfare agents of the Sarin or VX type [1,2], are of the greatest importance. Because of the toxicity and extent of use of cholinesterase inhibitors, close attention is being paid to their analysis [3,4,5]. There is a number of known physical and physicochemical instrumental methods used for the analysis of pesticides and chemical warfare agents in contaminated samples. However, their use is limited, due to the rather lengthy time of preparation of samples, complexity of the instrumentation, and high operational and financial costs [6,7,8]. One of the modern trends in the analysis of cholinesterase inhibitors is the development of analytical methods, which are characterized by simplicity, speed, high sensitivity, selectivity, and low cost. The biosensors that are best suited for these requirements are based on a cholinesterase reaction [9,10,11,12,13,14]. The principle of this reaction is the enzymatic degradation of the substrate (alkylcholine or alkylthiocholine) to obtain the appropriate acid or thiocholine, respectively, which can be detected by the appropriate methods and procedures. The simplest biosensors are based on the use of acid-based or coloured redox indicators, which allow the visual evaluation of the change in colour with the naked eye [15,16,17,18,19,20,21,22,23,24,25,26,27]. These types of biosensors are not primarily intended for quantitative determination of the analyte.

**Figure 1:**
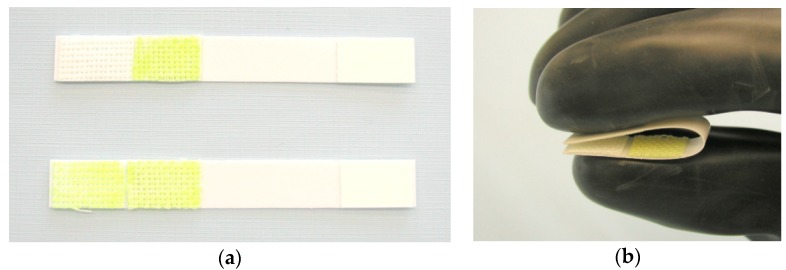
Biosensor Detehit: (**a**) appearance of the biosensor before and after use with a negative result; (**b**) the method of connecting the opposite zones after incubation.

**Figure 2:**
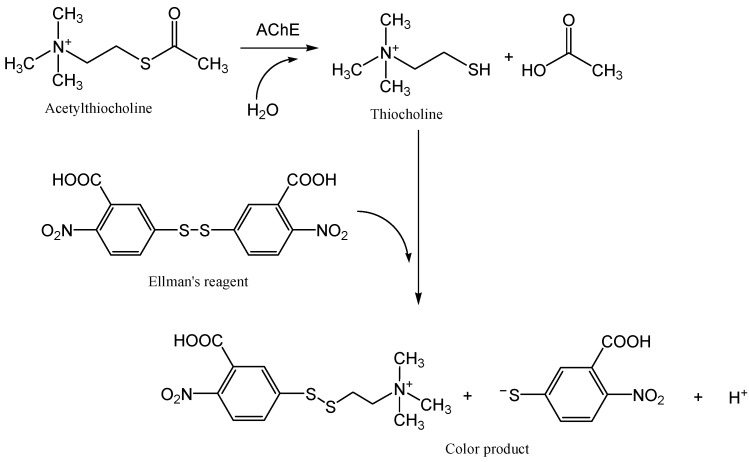
The schematic depiction of the analytical reaction in the biosensor Detehit.

An example of a simple biosensor based on visual evaluation of the colour change is the Czech product, Detehit, designed to detect chemical warfare agents of the Sarin and VX types in air, water, aqueous homogenates, food, soil, and on the surface of objects [28]. This is a detection ribbon based on special hardened polyvinylchloride, which contains a detection fabric with immobilized and stabilized AChE (acetylcholinesterase), a yellow standard, and a cellulose paper strip impregnated with ATChI (acetylthiocholine iodide) and Ellman’s reagent (Figure 1). The principle of detection is shown in Figure 2. Exposure of the biosensor is performed by exposing a moistened detection fabric to air, by immersing the fabric in contaminated water, or simply by pressing a wet detection zone against the test surface. The cellulose paper strip (Figure 1b) is pressed against the exposed detection fabric, and the change in colour of the detection fabric is then visually evaluated. If there is an absence of inhibitors, the original white detection fabric will change its colour to yellow (Figure 1a). If an inhibitor is present, the detection fabric remains white. The limits of detection depend on the type of analysis of the substance and on the exposure time.

The disadvantage of the Detehit biosensor is a less pronounced colour effect, especially under adverse lighting conditions. The aim of the work was to increase the intensity of the yellow colour by proposing a new substrate carrier made of glass nanofibres and using a chromogenic reagent (Ellman’s reagent). The function of this carrier was studied in comparison with the existing carrier: filter cellulose paper. To assess the characteristics of the new carrier in more detail, Ellman’s reagent was used in combination with the existing ATChI substrate and with an alternate BuTChI (butyrylthiocholine iodide) substrate. The function of the entire biosensor was tested with AChE and BuChE (butyrylcholinesterase).

## 2. Materials and Methods

### 2.1. Chemicals and Equipment

The following enzymes have been used: lyophilized horse plasma butyrylcholinesterase (BuChE) and lyophilized acetylcholinesterase (AChE), both supplied by Sigma-Aldrich; and acetylcholinesterase isolated from brain tissue (nucleus caudatus) of the species *Sus scrofa* f. domestica. Additionally, Ellman’s reagent (5,5′-dithiobis-(2-nitrobenzoic acid) was used, as well as acetylthiocholine iodide (min. 99%, ATChI), butyrylthiocholine iodide (min. 99%, BuTChI), corn dextran, Na_2_HPO_4_, KH_2_PO_4_ (all from Sigma-Aldrich, St. Louis, MO, USA), anion surfactant C12-14 alcohol 7EO (Enasol, Czech Republic), ethanol 99% (Penta, Czech Republic), and double-distilled water. As a carrier for the substrate and the reagent, we used MN GF-5 filter paper from glass fibres without a binder, with specific weight 85 g/m^2^ and a thickness of 0.4 mm (Macherey-Nage, Dueren, Germany), and filter cellulose paper with specific weight 85 g/m^2^ as a standard (Whatman, Kent, UK).

Physostigmine (98%, Sigma-Aldrich, St. Louis, MO, USA) and Sarin (O-isopropyl methylphosphonofluoridate, 94%, Military Research Institute, Brno， Czech Republic) were used as the inhibitors. Basic solutions, prepared by dissolving them in ethanol (0.01 g/10 mL), were diluted with water when necessary.

The measurement of the colour intensity was performed with a tristimulus colorimeter LMG 173 (Dr. Lange, Dusseldorf, Germany), SEM pictures were taken by a Tescan Vega-3 LMU electron microscope (Oxford Instruments, Abingdon, UK).

### 2.2. Preparation of the Biosensor

**Figure 3:**
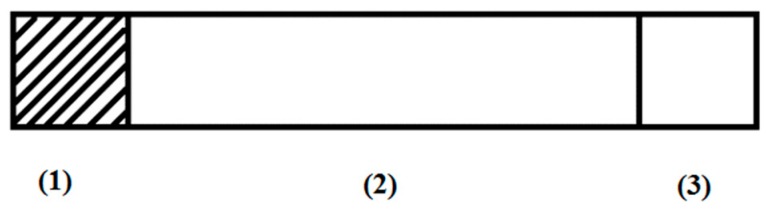
Diagram of the biosensor: (**1**) detection fabric with immobilized enzyme; (**2**) plastic strip; (**3**) carrier of substrate and indicator.

The biosensor consisted of a 10 cm long and 1 cm wide plastic strip, one end of which had an indication fabric (1 cm^2^) with an immobilized enzyme. At the other end it had the carrier (1 cm^2^) impregnated with a substrate and an indicator. A structure diagram of the biosensor is shown in Figure 3. The standard was not part of the biosensor, since it was not necessary to achieve work objectives.

The detection fabric with an immobilized enzyme (AChE tissue, AChE, or BuChE) was prepared by impregnating a white cellulose fabric with a solution containing an adequate amount of enzyme with total activity of 21 nkat/mL, 5% of dextran, and 2% of anionic tenside, in a phosphate buffer solution with pH 7.6. After the impregnation of the fabric, it was dried at a temperature of 25 °C for 24 h.

The glass and the standard cellulose papers were modified in accordance with the basic procedure used for the Detehit biosensor. Both carriers were impregnated with a solution of Ellman’s reagent (4.3 mmoL/L) and with a substrate (ATChI or BuTChI, both 6 mmoL/L) in ethanol. The indicator paper was dried at a temperature of 25 °C for 6 h.

### 2.3. Test of Functionality (Optimization of Experimental Conditions)

The testing (optimization) was focused on the following parameters: the influence of the carrier on the discolouration of the biosensor, the influence of the enzyme and the substrate being used (a combination of AChE(tissue)-ATChI, AChE-ATChI, and BuChE-BuTChI), the influence of the substrate concentration, the effect of the incubation period, stability, and other factors.

The system function and its colour response to the presence/absence of cholinesterase inhibitors were monitored visually and by using a tristimulus colorimeter. The tristimulus colorimetry is a type of reflex colorimetry (spectrophotometry), which is based on the CIE-L*a*b* colour system. In this system, L* is the neutral brightness axis, a* is the chromatic green-red axis (+a* red, −a* green), and b* is the chromatic blue-yellow axis (+b* yellow, −b* blue). In practice, the colour difference Δ*E* is often also used, which is defined by the Equation

Δ*E* = [(ΔL*)^2^ + (Δa*)^2^ + (Δb*)^2^]^1/2^,

where ΔL*, Δa*, and Δb* are the differences between individual values of L*, a*, and b* of the standard and the controlled colours. In our work, we have used the values on axis b* (designated as parameter b*) as the analytical signal.

A blank test (without inhibition) was performed as follows. The detection fabric was moistened with water and left for 1 min (to revive the enzyme), then the opposite ends of the biosensor were connected for 30 s. Then we monitored the discolouration of the detection fabric at regular intervals (1 min) with an immobilized enzyme, both using the tristimulus colorimeter and with the naked eye.

We carried out tests to monitor the impact of the inhibitor in the same way. The detection fabric with enzyme was moistened with 160 µL of physostigmine solution, and after the incubation time (1, 2, and 3 min), the opposite ends of the biosensor were connected for 30 s. The change of colour in the detection fabrics with enzyme were monitored on the tristimulus colorimeter and with the naked eye.

## 3. Results and Discussion

### 3.1. The Characteristics of the Carrier and Its Impact on the Intensity of the Achieved Colour

**Figure 4:**
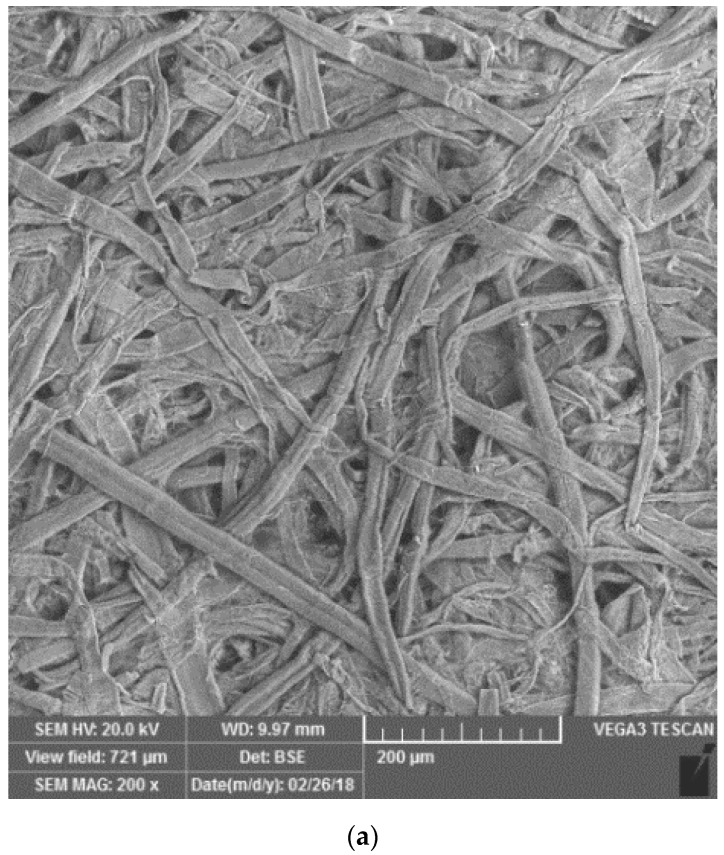

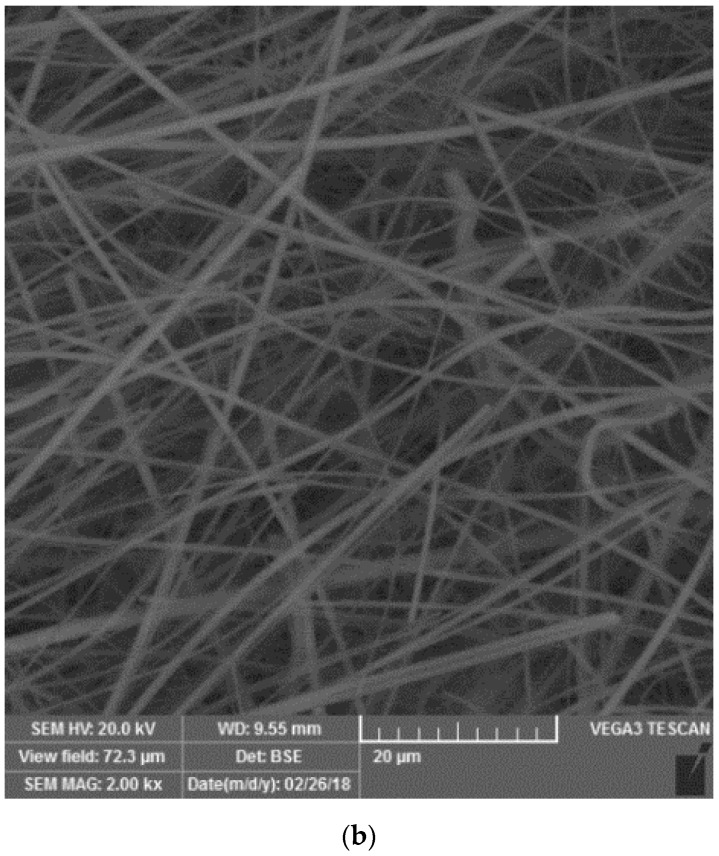
The SEM images with (**a**) the structure of the cellulose paper (resolution 200×) and (**b**) glass fibre paper (resolution 2000×).

The basic structure of the glass paper (for comparison, see also the structure of the cellulose filter paper used as a standard in the Detehit preparation), is shown in Figure 4, which was taken with SEM electron microscopy. The figure shows that the glass paper is made of fibres with nano dimensions.

**Figure 5:**
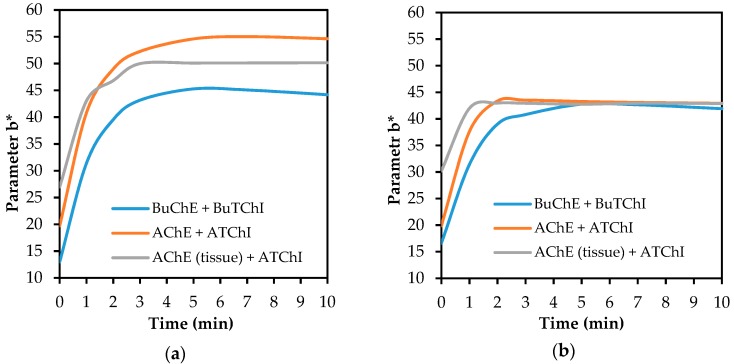
The development of the change in colour of the detection fabric (blank test), depending on the composition of the detection system with enzyme–substrate: (**a**) filter glass paper; (**b**) filter cellulose paper.

Primary attention has been paid to the impact of both carriers on the intensity of the colour of the detection fabric. As shown in Figure 5, the blank test proved that the paper made of glass nanofibres provides a better colour effect than the standard cellulose paper. For both carriers (for all tested combinations of enzyme-substrate), the colour intensity on the detection fabric was achieved sufficiently within 2 min and was appropriate even for a visual evaluation.

**Figure 6:**
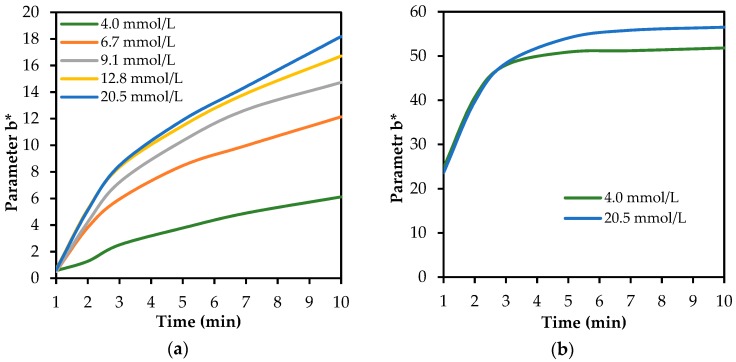
The influence of the ATChI substrate concentration on the change of colour in a fabric impregnated with AChE: (**a**) inhibited within 1 min with 10 µg/mL solution of physostigmine and (**b**) in the blank test.

The authors also investigated the dependence of the inhibitory effect on physostigmine on the required quantity of substrate. It was confirmed that the colour intensity increases in time with the increasing concentration of the substrate. The characteristic development of these dependencies (for the system AChE-ATChI) is illustrated in Figure 6. It seems that physostigmine acts as a competitive antagonist, and with the increasing concentration of the substrate, the physostigmine is displaced from the active centre of the inhibited enzyme. Relative standard deviations obtained with the tristimulus colorimeter had a maximum of 5%. The same deviations were reached with all other measurements.

### 3.2. The Influence of the Carrier on the Effectiveness of the Inhibition of Enzyme

**Figure 7:**
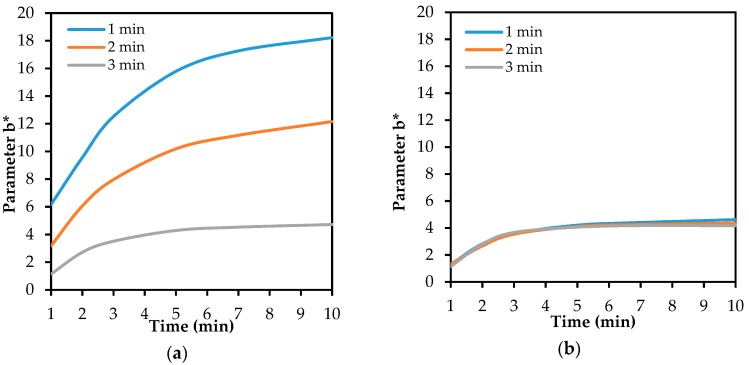
The influence of the incubation period on the inhibition of BuChE by physostigmine (10 µg/mL), and therefore on the discolouration of the detection fabric (carriers with BuTChI): (**a**) filter glass paper; (**b**) filter cellulose paper.

The enzyme inhibition with physostigmine showed that the two studied carriers had different behaviours. The results of the measurement of colour intensity indicate the different volumes of substrate and reagent, which are transferred onto the detection fabric with immobilized enzyme (Figure 7). It was obvious that, in the case of use of the cellulose paper with an incubation period of 1 min, the colour intensity was significantly lower than in the case of the glass paper. A lower colour intensity indicates that a substantially smaller substrate quantity has been transferred. This phenomenon (most pronounced for the system BuChE-BuTChI) resulted in less substrate being spread, which was reflected in a lower colour intensity due to a smaller quantity of reduced Ellman’s reagent. A prolonged incubation period, in the case of paper made of glass nanofibers, caused a higher degree of enzyme inhibition, while in the case of cellulose paper, no significant changes were observed. The results of measurements for the inhibition period of 3 min are comparable for both carriers.

### 3.3. The Effect of the Enzyme Being Used

**Figure 8:**
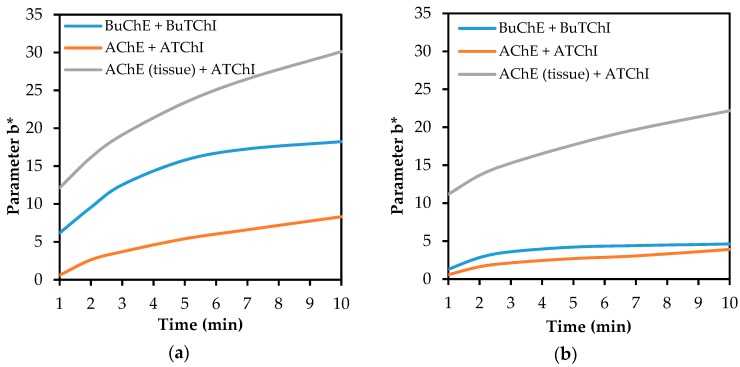
A comparison of the influence of the inhibition of enzyme by physostigmine (10 µg/mL) after 1 min incubation on the discoloration of the detection fabric in all tested enzyme systems, including substrate and carrier: (**a**) filter glass paper; (**b**) filter cellulose paper.

Although the Detehit preparation is based on AChE obtained directly from animal tissue, this work also verifies the commercially-available lyophilized AChE and BuChE. It was found that the disposition of physostigmine to inhibit these commercially available enzymes is higher than the disposition to inhibit the enzyme used as a standard. As it is shown in Figure 8, this was demonstrated for both of the investigated carriers. Lower sensitivity of the system to AChE isolated from animal brain tissue is probably caused by lower enzyme accessibility to the inhibitors of the physostigmine type. Physostigmine also shows better inhibitory capabilities at lyophilized AChE rather than at BuChE. The reason for the difference in the inhibition of these enzymes is probably a different compatibility of the active centres of enzymes with physostigmine.

### 3.4. Stability

**Figure 9:**
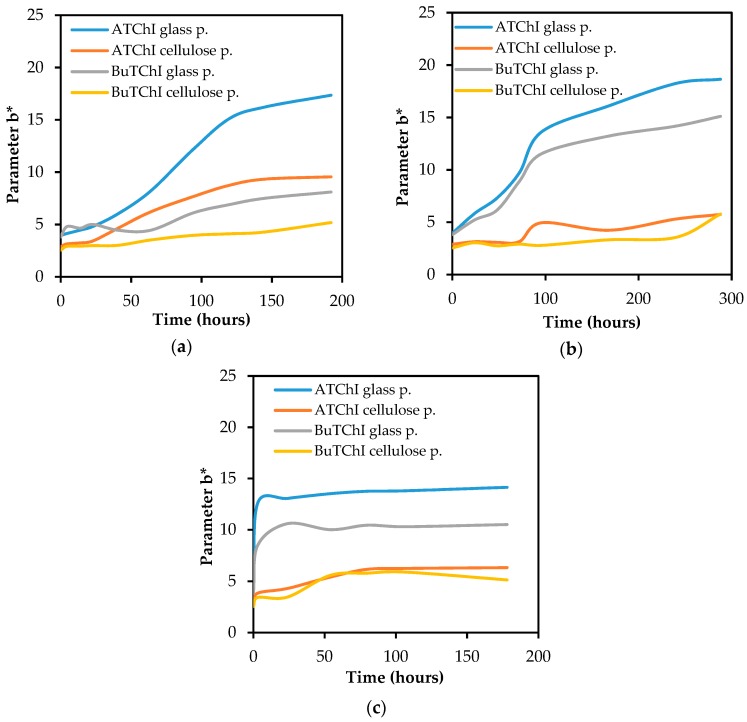
The stability of the substrate and Ellman’s reagent on a carrier: (**a**) in a dryer at 60 °C in a hermetically sealed glass container; (**b**) in the open air and light at room temperature; (**c**) loosely stored in a dryer at 60 °C.

**Figure 10:**
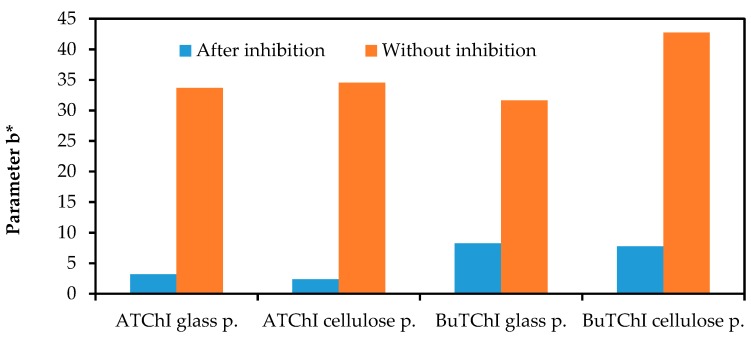
The functions of the biosensor after testing the thermal stability in a dryer in a container at 60 °C, with inhibition by physostigmine solution (concentration 100 µg/mL, incubation period 2 min, measured after 2 min).

Stability tests have been carried out in three modifications to impregnated carriers: (1) left in the open air, in light, and at room temperature; (2) stored loosely in a dryer at 60 °C; and (3) stored in a closed glass container in the dryer at 60 °C. Both carriers were impregnated with Ellman’s reagent in combination with ATChI or BuTChI, respectively. It was found by measuring the colour on the surface of the carriers by tristimulus spectrometry that the reagents on cellulose paper are more stable than those on the paper made of glass nanofibres. It is also evident that, under experimental conditions, the systems with BuTChI were more stable than systems with ATChI. Both of these conclusions are documented in Figure 9. For both carriers, the function of the biosensor after heat stress was verified with physostigmine. The systems with paper made of glass nanofibres provided more analytically appropriate results, i.e., greater differences between the activity of a non-inhibited and an inhibited enzyme (Figure 10).

### 3.5. The Limit of Detection

The aim of this work was not a correct determination (by objective methods) of the detection limit of the whole spectrum of potential inhibitors of cholinesterase. The intention of the experiment was only to verify whether the new carrier from glass nanofibres could give results comparable with the existing carrier, while using visual observation of the changes in colour. An organophosphorus chemical warfare agent—Sarin—was used for this purpose. Sarin belongs among the most powerful of cholinesterase inhibitors. The specified detection limit corresponded to the concentration of Sarin, for which the detection fabric remained white even after 2 min of contact with the paper carrier of the substrate and the chromogenic reagent (the blind sample was yellow). Comparable results were achieved for both carriers; however, the paper from glass fibres provided a much more reliable visual evaluation. The limit of detection was significantly influenced by the enzyme used. In the case of lyophilized AChE, the value of the detection limit was 0.0005 µg/mL; in the case of BuChE, it was higher by about one order of magnitude, i.e., 0.005 µg/mL.

## 4. Conclusions

For a simple biosensor of cholinesterase inhibitors, a new carrier of substrate and indicator was designed, which is made of paper made from glass nanofibres. During the comparative tests with a standard carrier, which was used at the manufacture of Detehit biosensor (a cellulose filter paper), it was found after impregnation that the glass paper absorbs a greater quantity of substrate and indicator. Glass paper is able to release these chemicals more easily than cellulose paper, and it allows a better transition of these chemicals to the indication fabric with an immobilized enzyme. This in turn allows more intense discoloration of the detection zone of the biosensor, which has a beneficial effect as an easier and more reliable assessment of the test results, including their visual evaluation with the naked eye.
